# Self-Balancing Position-Sensitive Detector (SBPSD)

**DOI:** 10.3390/s150717483

**Published:** 2015-07-20

**Authors:** Ryan Porrazzo, Leigh Lydecker, Suhasini Gattu, Hassaram Bakhru, Natalya Tokranova, James Castracane

**Affiliations:** Colleges of Nanoscale Science and Engineering, SUNY Polytechnic Institute, 257 Fuller Road, Albany, NY 12222, USA; E-Mails: llydecker@albany.edu (L.L.); Sgattu@sunycnse.com (S.G.); HBakhru@sunycnse.com (H.B.); ntokranova@sunycnse.com (N.T.); JCastracane@sunycnse.com (J.C.)

**Keywords:** PSD, back-to-back connected photodiode, linear position sensor, optical position sensor

## Abstract

Optical position-sensitive detectors (PSDs) are a non-contact method of tracking the location of a light spot. Silicon-based versions of such sensors are fabricated with standard CMOS technology, are inexpensive and provide a real-time, analog signal output corresponding to the position of the light spot. An innovative type of optical position sensor was developed using two back-to-back connected photodiodes. These so called self-balancing position-sensitive detectors (SBPSDs) eliminate the need for external readout circuitry entirely. Fabricated prototype devices demonstrate linear, symmetric coordinate characteristics and a spatial resolution of 200 μm for a 74 mm device. PSDs are commercially available only up to a length of 37 mm. Prototype devices were fabricated with various lengths up to 100 mm and can be scaled down to any size below that.

## 1. Introduction

Position-sensitive detectors are used to track the position of a light spot in one or two dimensions [1]. Some common applications of PSDs is range determination by triangulation using a laser and obstacle detection [2,3]. Only 1D PSDs will be discussed in this work. There are two main classes of optical position sensors, or position-sensitive detectors (PSDs). The first type is composed of discrete photo sensor elements that provide position information in the form of a digital output (e.g., CCDs [4] or linear array of discrete photodiodes). The second type is a sensor with a continuous photosensitive surface that provides position data in the form of an analog output (e.g., Lateral effect PSD [5,6]).

The lateral photo effect was officially discovered by Wallmark in 1957, but the effect was originally described by Schottky in 1930 [1,7]. The effect was further developed by Lucovsky in 1960, and has since been developed by other research groups [8,9,10]. The basic structure of a lateral effect PSD is a line composed of a through-wafer photodiode with a contact on either side of the top of the line, and a common contact along the backside of the device. The device is reverse-biased in order to increase the width of the depletion region of the photodiode, and thus the sensitivity to light. Carriers are generated by incident light in the depletion region of the photodiode, and drift through the bulk of the device due to a junction field induced by the applied bias. The generated current is then divided between the two top contacts as a function of the resistance between the incident light spot and each respective contact. Using the ratio of these two currents, the position sensitive detector reveals the median of the incident light spot that is independent of the size or shape of the light spot within the active area.

Improvement of thin film technology has allowed for the creation of large area 1D thin film PSDs along with the ability to create nanostructured silicon for applications in solar cells, position sensors, and thin film transistors [11,12].

Lateral effect position-sensitive detectors (LEPSDs) provide an analog output, offer high resolution, and are a relatively low cost, accurate method of sensing position using standard CMOS processing techniques [13,14]. However, packaging a sensor that needs a backside contact requires a higher packaging cost. If a sensor could have all contacts on one side of the substrate, then the cost of the sensors and their packaging could be reduced by eliminating all backside processing and associated costs. Lastly, minimization of required readout circuitry is desired. Analog output position sensitive detectors typically require less signal processing circuitry than a digital output position sensor. However, developing a sensor with a direct readout from the sensor itself eliminates the need for any external circuitry, which makes using the device for various applications easier and less expensive. Consideration of these factors led to the development of a new type of sensor that will be described in this work.

### Device Operation

**Figure 1:**
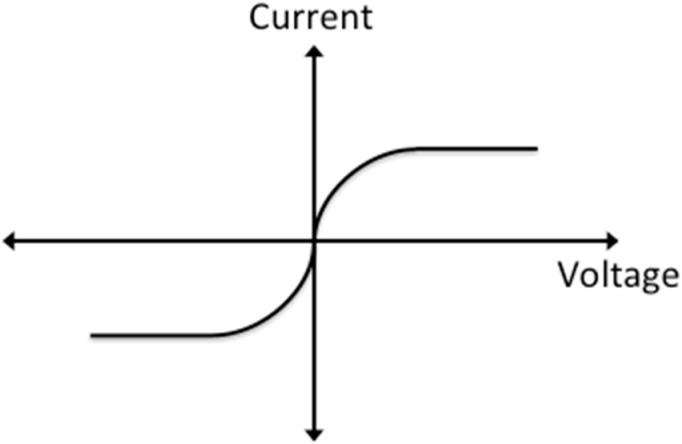
I-V curve of n-p-n junction.

The self-balancing position-sensitive detector (SBPSD) consists of two parallel doped lines in a substrate of the opposite doping type with a metal line connecting the contact pads on top of both devices. The line where voltage is applied shall be known as the distributed bias line while the line which the final potential is measured is called the floating line. In our case, two n-type lines were produced in a p-type substrate. Each line is resistive, based on the doping concentration used. The two lines form a linear back-to-back photodiode pair from the n-p-n junction along the device, resulting in the characteristic I-V curve shown in Figure 1. Because the photodiodes are connected back-to-back, one of them is reverse-biased and the other is forward-biased. The reverse bias determines the current flowing inside the device.

The floating line must be measured with a high input resistance device in order to maintain current flow inside the device [15]. Once illuminated, one of the diodes will be split into a part that is reverse-biased on the right side of the illuminated spot, while the left side will be forward-biased. The second diode will then be forward-biased on the right side of the illuminated spot while the left side will be reverse-biased. The potential difference across the n-p-n junction determines the state on the diode, which part of the diode is reverse-biased, which part of the diode is forward-biased, and the resulting direction of current flow. When a light spot is incident on the pair of back-to-back connected photodiodes, the reverse-biased part of the diode generates current. This current travels freely through the forward-biased part of diode, accumulating charge on the floating line and thus changing its potential [16]. The biased line maintains a constant linear potential distribution.

**Figure 2:**
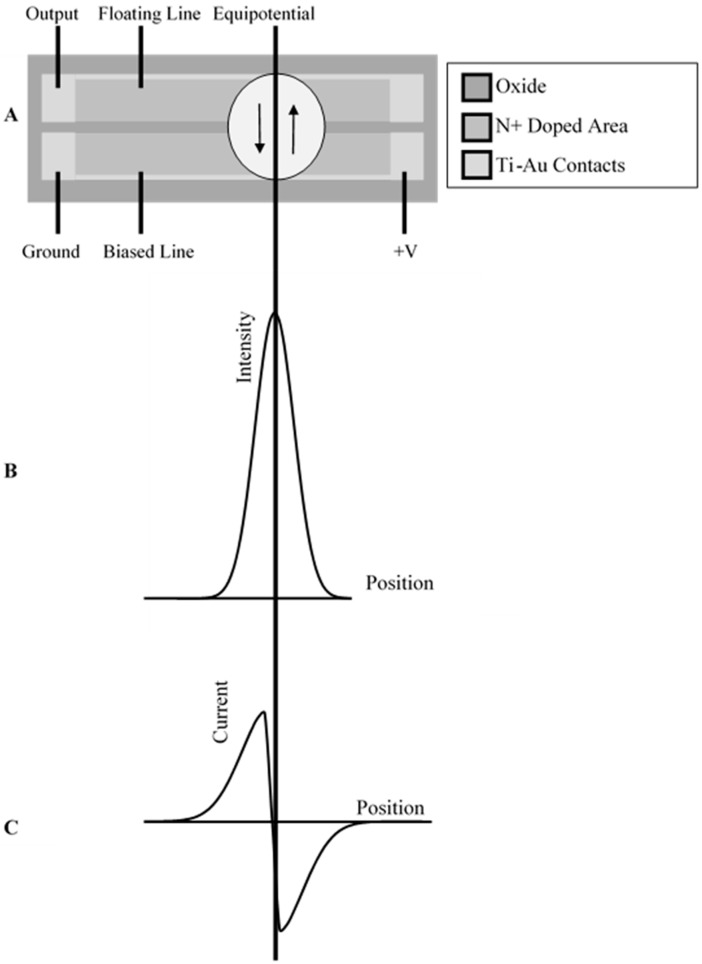
(**A**) Illustration of direction of current flow under illuminated light spot at the equilibrium position; (**B**) spatial distribution of the intensity of the light spot; (**C**) current produced in the device upon illumination in equilibrated state.

Potential difference between lines at each point across the device determines current direction at each location. The illuminated light spot creates a flow of current between the lines as shown in Figure 2. The generated currents automatically balance their flows around the median of the light spot. The equipotential is the position where the potential values of two lines are the same. At equilibrium the median of the light spot and the device equipotential are located at the same position, allowing for determination of the spot position relative to the device. The flow of current between the two lines causes the potential on the floating line to equilibrate to the same value as that of the biased line at the median of the light spot. At the equilibrium, the currents on both side of the center of the light spots are equal [17].

A critical figure of merit for photodiodes is their responsivity, which is the ratio of the optical generated current to the input power of the light, as shown in Equation (1) [18].

(1) $$ℜ=\frac{I_{op}}{P_{opt}}$$

The responsivity is given in units of amps per watt (A/W). Typical responsivity values of silicon photodiodes at 623 nm is ~0.4 A/W [19].

**Figure 3:**
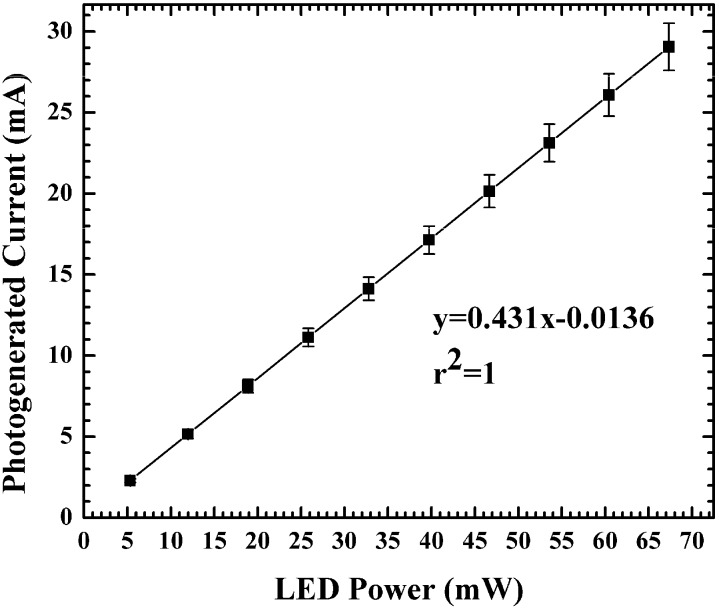
Photogenerated current from SBPSD as a function of incident LED power, showing the device responsivity in A/W as the slope of the curve.

In order to determine the responsivity of the position sensitive detectors presented, I-V curves were measured at various LED intensities controlled by the applied current as well as with no illumination. The photogenerated current was obtained by subtracting the measured dark current from the illuminated response. The light intensity was measured using calibrated photodiodes inside a Newport 1815-C Power Meter. The spot used for measurement was completely inside the photosensitive area of the device, the same as in the calibrated photodiode. The responsivity of the prototype detectors in this work was determined to be 0.43 A/W after averaging measurements on three devices, as shown in Figure 3, which compares favorably with commercially available photodiodes illuminated with a 623 nm wavelength LED.

## 2. Experimental Section

6″ (100) p-type silicon wafers with a resistivity of 80–130 ohm-cm, a thickness of 675 μm, and 500 nm of oxide on the surface were used for the device fabrication. First, alignment marks were patterned onto the front of the substrate using standard contact photolithography processing. The alignment marks were etched through the SiO_2_ into the silicon wafer itself, providing a permanent reference feature for future alignment.

**Figure 4:**
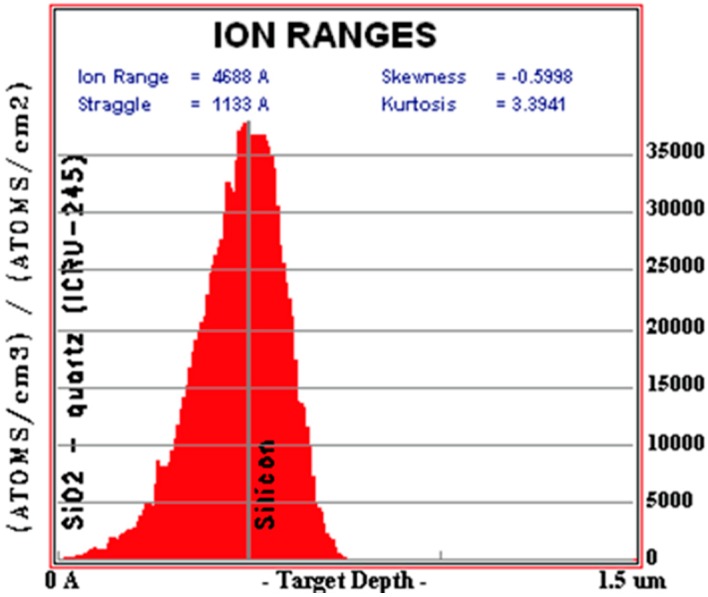
Simulation of hosphorous ion implantation through surface oxide using SRIM software.

The main doped device lines were then defined onto the SiO_2_ with standard contact photolithography processing, leaving only the desired doping area exposed. The parallel device lines were then doped with phosphorous through the oxide using ion implantation. The implant energy was 350 keV and the dose was 1.67 × 1016 ions/cm^2^, as shown in Figure 4, simulated by SRIM (Stopping Range of Ions in Matter) software. After the implant, the photoresist mask was stripped, and the wafers were annealed in argon at 1000 °C for 1 min using a rapid thermal anneal process to thermally activate dopants and fix damage in the crystal lattice.

Openings in the SiO_2_ were patterned using contact photolithography and etched using buffered oxide etchant (Transene Company, Inc. Danvers, MA, USA) in order to allow direct contact to each of the doped silicon lines. The electrode metals, 200 Å of titanium followed by 1500 Å of gold, were then deposited onto the substrate, and subsequently patterned to form the electrical device contacts. Upon completion, the devices were sent for dicing.

In order to analyze the influence that the width of the separation line between the doped areas has on device performance, devices were fabricated at lengths of 37 mm, 74 mm and 100 mm.

Measurements were performed directly on the device with no external electronic circuitry. Device characterization was done by incrementally moving a light spot along the device using a LabVIEW program that moved a microscope head attached to a Cascade Microtech Alessi REL-6100 programmable probe station to incrementally scan the length of the device with the light spot focused by a microscope. A Keithley 238 source measure unit (SMU) was used to read and record the output voltage while a second Keithley 238 SMU was used to apply the 0 to 5 V bias. Before measurements were performed, the light spot was first centered to fit the entire device width. However, if the device were misaligned or the light spot did not cover the whole device, it would cause asymmetrical illumination on the device, which would affect the resulting coordinate plot. The response of the device to a moving light spot is illustrated by the coordinate plots to follow in the paper. The coordinate characteristic of the PSD is a plot of light spot position *vs.* the output voltage. Self-balancing position sensors were fabricated, packaged and tested.

## 3. Results and Discussion

**Figure 5:**
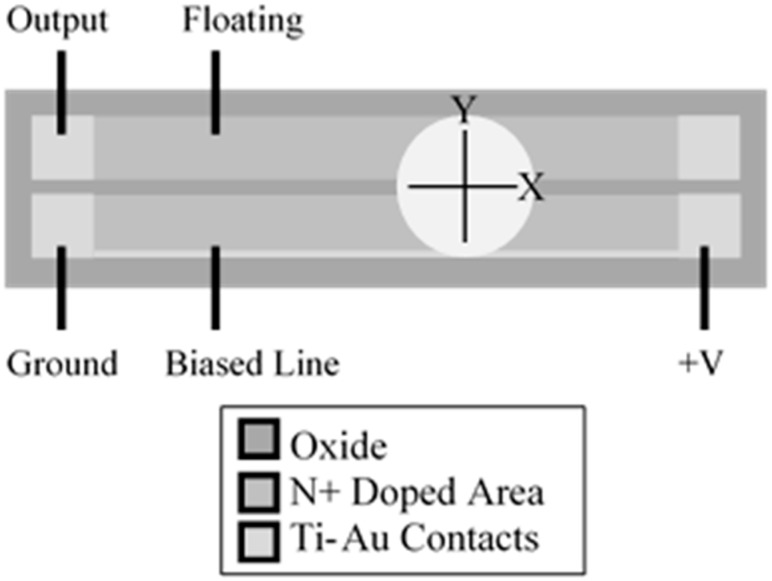
Schematic drawing of light spot position.

**Figure 6:**
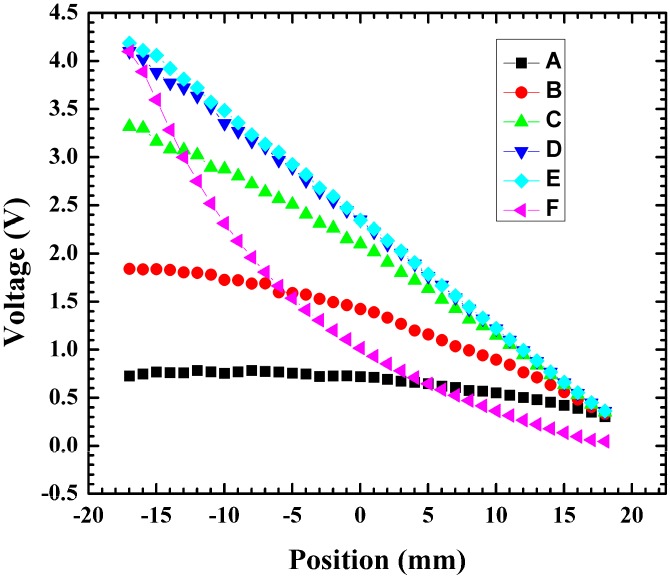
Output voltage *vs.* position measurements for various light positions (A = −0.4 mm, B = −0.2 mm, C = 0 mm, D = 0.2 mm, E = 0.4 mm, F = 0.4 mm). At positions A–E the 0–5 V bias was applied to the bias line and the floating potential measured on the floating line. For position F, the 0–5 V bias was applied on the floating line and the floating potential measured on the biased line.

To investigate this effect, 37 mm-long devices fabricated with a metal line connecting the contact pads for doped lines while bare silicon connected the other doped lines were used for testing (Figure 5). Testing was performed by moving the light spot incrementally by an offset in the vertical direction by 0.2 mm from the center, and the same measurement was performed. The light spot was further offset 0.2 mm to the left for a total of 0.4 mm from the center light spot and a coordinate plot was measured (Figure 5). The same measurement was then performed on the bottom portion of the device at 0.2 mm and 0.4 mm from the center. In Figure 6, points E and F are positioned identically with reference to the light spot, with the only difference being which line the 0 to 5 V bias was applied to. Point E clearly demonstrates that application of a bias onto the metal line achieved a linear profile compared to the curved profile in point F, where the bias was placed on the side with no metal line connecting the contacts. A metal line connecting the contact pads across the device led to fewer losses when compared to a nonmetal line. Lateral displacement in the light spot from the nonmetal to the metal causes the resistance in the nonmetal to decrease, leading to a more linear coordinate plot.

I-V measurements were performed to further understand changes in the coordinate characteristic due to variation in spot location. Measurements were performed using a light spot with a Gaussian profile which led to a non-symmetric volt-ampere characteristic for these devices with respect to the position of the light spot across the device. I-V curves were measured by sweeping from −2 to 2 V and recording the corresponding current. If the light spot does not illuminate part of both lines identically, the I-V curve changes in response. This effect was confirmed by measuring the 37 mm-long SBPSD with 50 micron spacing between the doped lines by first placing the light spot such that it covered the whole device width and measuring the IV response.

**Figure 7:**
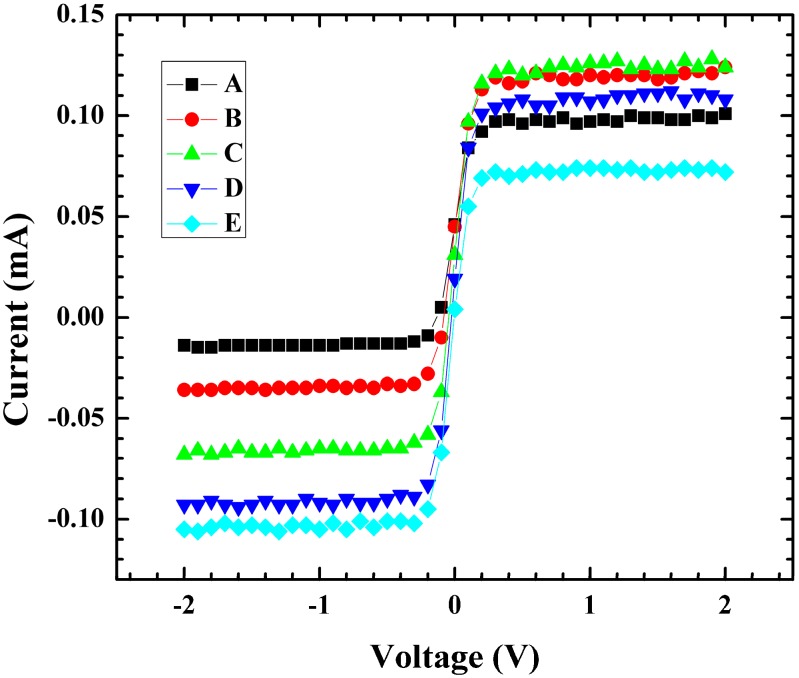
Output voltage *vs.* current measurements for various light positions (A = −0.4 mm, B = −0.2 mm, C = 0 mm, D = 0.2 mm, E = 0.4 mm).

The location of the light spot was changed similarly to Figure 5, by moving the spot position by 0.2 mm and 0.4 mm in the Y and −Y direction and an I-V curve was measured at each spot location. In the resulting I-V curves (Figure 7), the positive saturation current decreased for all measurements that were not in the center of the device. For the negative saturation current, a noticeable difference was observed; depending on which side of the device the light spot was moved, the current changed in response. When the light spot was offset towards the biased line, the saturation current was more negative (lower) than at the center light position. When the light spot was offset towards the floating line the current was less negative (higher) than the center light position. This change in the I-V response led to a corresponding change in the coordinate characteristic for these devices. The performance measurements confirmed the importance of light positioning on the photosensitive surface of the device for accurate coordinate determination.

**Figure 8:**
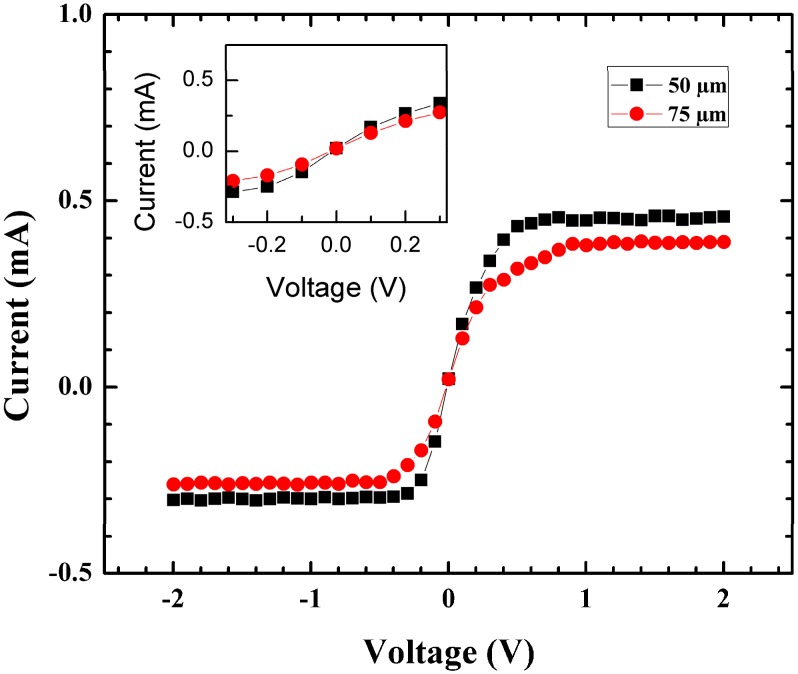
I-V curve of 37 mm SBSPD with 50 μm and 75 μm spacing between doped lines. Inset from −0.3 to 0.3 shows the resistance changes when varying the distance between doped lines.

Investigations were performed to determine how the distance between doped lines would affect the slope of the I-V curve. 37 mm devices were fabricated with a distance of 50 µm and 75 µm between the doped lines. Various distances between the doped lines will result in different values in resistance between lines and changes in the slope of the transition part between saturation regions of I-V curves as can be seen in the inset in Figure 8.

The distributed bias line is the doped silicon and its resistance can be changed by light illumination. Results of the above investigation regarding changes in the coordinate plot based on light spot displacement led to the development of a PSD with dual metal lines as shown in Figure 2a. The addition of dual metal lines eliminated the effect of lateral displacement in the light spot. The influence of resistance change due to photosensitivity on SBPSD coordinate response was studied. The coordinate characteristic of the device and its resistance along the biased line were measured to determine the effect of resistance change on the linearity in the coordinate plot. Two types of devices were used for this measurement. The devices that were used for this measurement are the dual metal line 100 mm length devices. The metal used for the contact pads is 1500 Å of Au on top of 500 Å of Ti. In one device the contact pads were covered with chemically resistant tape and the Au was etched, leaving only Ti on the line connecting the device. The device with only Ti across the device had a maximum resistance of 26.8 kΩ near the side where 5 V was applied compared to the device with Au-Ti, yielding a maximum resistance of 163.7 Ω.

**Figure 9:**
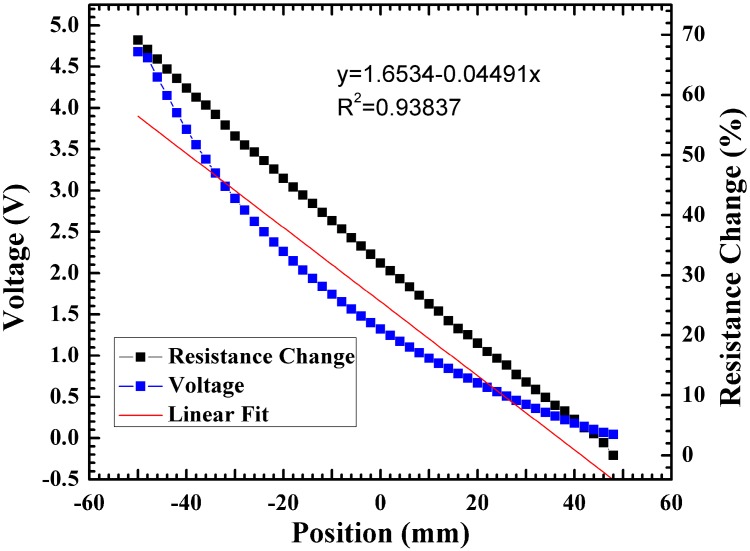
Coordinate plot and resistance change for a 0-to-5 V bias applied to the Ti metal line.

A 0-to-5 volt bias was first applied across the Ti line. Using this bias a coordinate plot for voltage and resistance change *vs.* position were measured, and the resistance was calculated by Ohm’s law. A significant resistance change (~70%) led to a non-linear, non-symmetric coordinate plot (R^2^ = 0.9387) (Figure 9).

**Figure 10:**
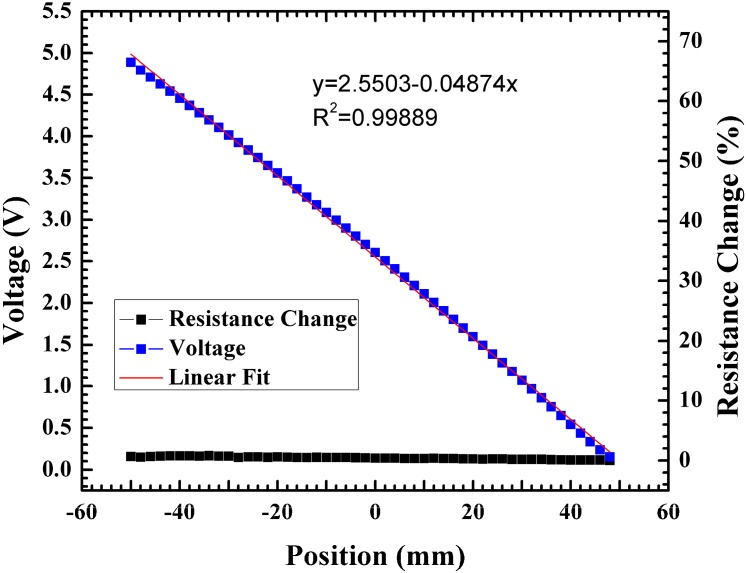
Coordinate plot and resistance change for a 0-to-5 V bias applied to the Ti/Au metal line.

The 0-to-5 V bias was then applied to the Au-Ti line. Using this bias condition, a coordinate plot for voltage and resistance change *vs.* position was measured. It was observed that having a low resistance led to a linear symmetric coordinate plot. The resistance was essentially constant across the device with an overall resistance change of 0.2%. The application of the bias to the device with the lower resistance led to a linear symmetric coordinate plot and a significantly lower resistance change as seen in Figure 10. For greater device lengths (>37 mm), dual metal lines are needed to achieve a linear coordinate plot due to the large non-uniformity in the potential distribution across the floating line that occurs when there is no metal line connecting the contacts.

**Figure 11:**
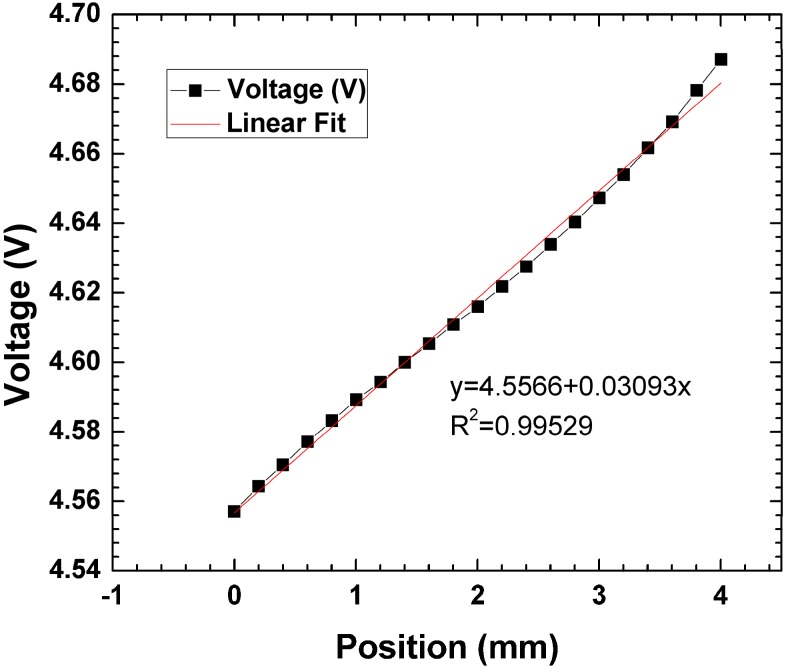
Coordinate plot over a 4-mm distance with 200 μm.

The theoretical spatial resolution of a PSD is the minimum detectable light spot displacement. For a 1-D PSD, as the active area increases, the position resolution decreases. For an active area of 6 mm, 12 mm and 37 mm, the corresponding position resolution is 0.2 µm, 0.3 µm and 2.8 µm respectively [20]. The theoretically smallest detectable position displacement can be expressed as

(2) $$∆x=L\times \frac{I_{n}}{I_{Tot}}$$

where L is the length of the detector, I_Tot_ is the total photocurrent from all contacts, and I_n_ is the noise current [13]. The theoretical spatial resolution for a 74 mm device was determined to be approximately 200 µm using Equation (2) in conditions where the photogenerated current exceeds the dark current by approximately 300 times. Theoretical spatial resolution was then verified by moving the microscope head on the probe station in 200-µm steps and the output voltages were recorded (Figure 11). A plot of voltage *vs.* position shows a highly linear coordinate plot (R^2^ = 0.99529) over a 4-mm distance.

**Figure 12:**
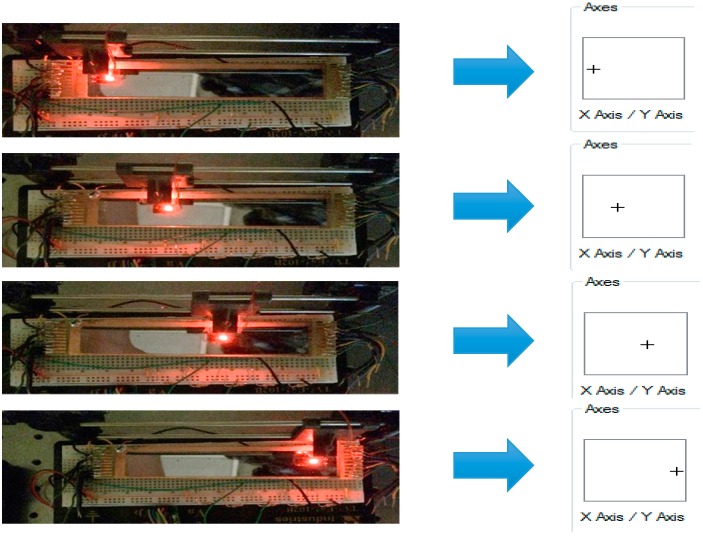
Demonstration of computer interfacing with SBPSD using built-in game controller configuration calibration settings.

A symmetric and linear coordinate plot is necessary for operation of the self-balancing position-sensitive Detector. The sensor’s operation was tested using Microsoft Windows built-in game controller configuration software. This configuration software allows for the user to set a voltage value inside a defined range to a light spot position along a particular axis. In this case, the output voltage varies with the movement of the LED and is defined as movement on the x-axis. The operation of the sensor is demonstrated in Figure 12.

## 4. Conclusions

This paper discusses a new approach to creating self-balancing position-sensitive detectors. The effect of resistance change on the linearity of the coordinate plot was investigated by comparing plots of voltage *vs.* light spot. Prototype devices were fabricated with lengths of 37 mm, 74 mm and 100 mm, respectively. Experimental measurements showed a linearity R^2^ = 0.9989 and a spatial resolution of 200 µm for a 74-mm device in conditions where the photocurrent is 300 times greater than the dark current. A new type of position-sensitive device with linear coordinate characteristics requiring no external circuitry or backside contacts was fabricated and tested, and the performance characteristics of these devices were established, paving the way for ultimate integration into the target product as well as various other applications.
